# Genetic diversity and natural selection analysis of *VAR2CSA* and *vir* genes: implication for vaccine development

**DOI:** 10.1186/s44342-024-00009-0

**Published:** 2024-07-15

**Authors:** Joseph Hawadak, Aditi Arya, Shewta Chaudhry, Vineeta Singh

**Affiliations:** 1 ICMR-National Institute of Malaria Research (NIMR), Delhi, 110077, India; 2 Academy of Scientific and Innovative Research (AcSIR), Ghaziabad, 201002 India

**Keywords:** Variable surface antigens, *Plasmodium* interspersed repeat, Genetic diversity, Natural selection

## Abstract

Variable surface antigens (VSAs) encoded by *var* and *vir* genes in *Plasmodium falciparum* and *Plasmodium vivax*, respectively, are known to be involved in malaria pathogenesis and host immune escape through antigenic variations. Knowledge of the genetic diversity of these antigens is essential for malaria control and effective vaccine development. In this study, we analysed the genetic diversity and evolutionary patterns of two fragments (DBL2X and DBL3X) of *VAR2CSA* gene and four *vir* genes (*vir* 4, *vir* 12, *vir* 21 and *vir* 27) from different endemic regions, including Southeast Asia and sub-Saharan Africa. High levels of segregating sites (S) and haplotype diversity (Hd) were observed in both *var* and *vir* genes. Among *vir* genes, *vir* 12 (*S* = 131, *Hd* = 0.996) and *vir* 21 (*S* = 171, *Hd* = 892) were found to be more diverse as compared to *vir* 4 (*S* = 11, *Hd* = 0.748) and *vir* 27 (*S* = 23, *Hd* = 0.814). DBL2X (*S* = 99, *Hd* = 0.996) and DBL3X (*S* = 307, *Hd* = 0.999) fragments showed higher genetic diversity. Our analysis indicates that *var* and *vir* genes are highly diverse and follow the similar evolutionary pattern globally. Some codons showed signatures of positive or negative selection pressure, but *vir* and *var* genes are likely to be under balancing selection. This study highlights the high variability of *var* and *vir* genes and underlines the need of functional experimental studies to determine the most relevant allelic forms for effective progress towards vaccine formulation and testing.

**Supplementary Information:**

The online version contains supplementary material available at 10.1186/s44342-024-00009-0.

## Introduction

Among the five malaria-causing species in humans, *Plasmodium falciparum* and *Plasmodium vivax* account for about 99% of malaria cases worldwide [1]. The last 10 years have seen an increase in reports of severe *vivax* malaria even though *P. falciparum* remains associated with highest morbidity and mortality [1]. *Plasmodium* success and persistence in endemic areas are mostly attributed to the extreme genetic diversity of parasite surface antigens and lack of effective vaccine [2, 3].

*Plasmodium* genomes contain multigene families located on telomeric and sub-telomeric portions of chromosomes encoding for variable surface antigen (VSAs). The multigene *var* family in *P. falciparum* encode for *P. falciparum* erythrocyte membrane protein 1 (PfEMP1), one of the major blood stage surface antigens. Each *P. falciparum* parasite contains about 60 *var* genes with mutually exclusive expression [4]. In other *Plasmodium* species, including those infecting rodents and humans, the orthologue family of *var* gene called *Plasmodium* interspersed repeat (*pir*) represents the largest multigene family known so far [5]. The *pir* gene is named *vir* in *P. vivax*, *kir* in* Plasmodium knowlesi*, *cyir* in *Plasmodium cynomolgi* and *bir* and *yir *in *Plasmodium berghei* and *Plasmodium yoelii, *respectively. The number of *pir* genes varies considerably between species, ranging from 134 in *P. berghei* to 1949 in *Plasmodium ovale curtisi* [6, 7]; however, they are less well-characterised because of their considerable antigenic diversity and challenges with in vitro culture long-term maintenance. Whole-genome analysis of *P. vivax* revealed that *vir* genes are more abundant than *var* genes and not related in terms of gene and protein structures but speculated to play similar function in disease pathogenesis [5, 6, 8]. Unlike the *var* genes, several *vir* genes express simultaneously in the same parasite at the blood stage [6, 7].

The relationship between VSAs and malaria pathogenesis has long been studied as a potential target for malaria vaccine development [4, 5, 9–11]. These genes can be viewed as promising vaccine candidates. Antigenic diversity involving differential expression of *var* genes is a key factor underpinning transmission dynamics within and between human hosts due to immune escape [3, 4]. Similar to *var* genes, *vir* multigene superfamily has been reported to be associated with the activation of the immune system and cytoadherence to endothelial cells and subsequently could induce the natural acquisition of antibodies after infection [11–13]. Transcription of DC8 and DC13 in the upregulated and specific domains of *var* proteins has been shown to be associated with cerebral malaria [14]. The *var* gene family also contribute to specific malaria pathology, such as brain swelling, cerebral malaria-positive retinopathy, severe malaria anaemia and respiratory distress [15]. In addition, there are several *var* genes with the same binding phenotype, allowing the parasite to maintain its adhesion to particular receptors despite recognition by antibodies [15]. For instance, VAR2CSA proteins bind to chondroitin sulphate A, a glycosaminoglycan expressed on placental syncytiotrophoblasts, via the DBL2X domain, resulting in placental sequestration of infected red blood cells (RBCs) contributing to poor birth outcomes [16]. This evidence underlines the important role played by *var* genes in the pathogenicity of *P. falciparum* malaria. In *P. vivax*, numerous *vir* genes are also speculated to be involved in cytoadherence. Transgenic-infected *P. falciparum* cells expressing VIR14 have been shown to bind to ICAM-1 and human spleen fibroblasts, as well as lung and brain endothelial cells in vitro [12, 17, 18]. Speculation remains open as to whether VIR antigens contribute to immune evasion as is the case for VSA in *P. falciparum* or whether other VIR and non-VIR proteins are involved in *P. vivax* cytoadherence and malaria pathogenesis.

Despite the fact that genes encoding for VSAs have been extensively studied, results show significant genetic diversity, notably in *var* and *vir* families in *P. falciparum* and *P. vivax*, respectively [10, 13, 19–21]. To assess the global genetic diversity and natural selection process operating in these genes, we analysed the following: (i) *VAR2CSA*, a dominant *var* gene well-characterised in vivo and expressed by *P. falciparum* infecting pregnant women, constituting the most promising *var* gene candidate vaccine and (ii) the most studied *vir* genes (*vir* 4, *vir* 12, *vir* 21 and *vir* 27) in global *P. falciparum* and *P. vivax* population, respectively. This study was carried out with the aim to understand the evolutionary dynamics of these genes and describe their distribution pattern globally. The knowledge of existing genetic diversity in these genes will be helpful in determining the potential targets for vaccine candidates for malaria species.

## Methods

### Sequences data set

All sequences included in this article are from our previous reported studies or downloaded from GenBank database. Eight-hundred and fifty-one *VAR2CSA* sequences (DBL2X and DBL3X domains) from Kenya, Colombia, Malawi, Mozambique, Benin; Gambia, Ghana, Mali, Nigeria, Tanzania, Senegal, Uganda, Republique Democratic of Congo (RDC) and Papua New Guinea (PNG) were analysed. A total of 293 *vir* sequences including *vir* 4, *vir* 12, *vir* 21 and *vir* 27 from India, Korea and Malaysia were also analysed. Those data previously analysed and published elsewhere are re-analysed in this study with a different approach and new tools (Supplementary Table 1).

### Genetic diversity and natural selection analysis

Sequence diversity was determined by calculating pairwise nucleotide diversity (π and Θω), and number of segregating sites (S), number of haplotypes (H), haplotype diversity (Hd) and average number of nucleotide differences (k) were computed using DnaSP6 software. The same software was used to perform Tajima’s D test on *var* and *vir* sequences to determine whether these genes were under random or nonrandom evolutionary process.

Maximum likelihood methods and Bayesian approaches were used to evaluate the effect of natural selection operating in *vir* and *var* genes. The Fast Unbiased Bayesian Approximation (FUBAR), mixed effects model of evolution (MEME) and fixed effects likelihood (FEL) methods were applied to provide additional support to the detection of sites evolving under positive or negative selection. The FUBAR, MEME and FEL were used to explore sites subject to positive diversifying selection, while signature of negative selection was detected by FUBAR and FEL methods only. All analysis was run in Datamonkey server with default parameters. The best-fitting nucleotide substitution model was determined through the automatic model selection tool. The amino acid codon under selection pressures either with threshold *p*-values ≤ 0.05 in case of FEL and MEME or posterior probability ≥ 0.95 for FUBAR were considered as statistically significant.

Entropy scores were also used to quantify amino acid sequence variation by using Entropy-One tool (https://www.hiv.lanl.gov/content/sequence/ENTROPY/entropy_one.html) with default parameters. Relative entropy scores (0 to 1) were calculated by comparing the amino acid probability distribution for each column of the multiple sequence alignment with that of the background distribution. Positions where all amino acids are identical are considered to have minimal positional entropy, i.e. 0. Conversely, positions where all amino acids appear at equal frequencies are considered to have maximum positional entropy with a value of 1.

### Recombination analysis

Seven methods including RDP, GENECONV, BootScan, Chimaera, MaxChi, SiScan and 3Seq were performed with default parameters using recombination detection program (RDP) programme v4.101 to account for potentially confounding effects of recombination in the inference of selection. Probable recombination event and their localization, recombinants and likely parental sequences were explored. Recombination events supported by at least five detection methods were considered after Bonferroni correction at a *p*-value ≤ 0.05. Recombination breakpoints identified were further re-evaluated with the genetic algorithm for recombination detection (GARD) implemented in Datamonkey server.

## Results

Two fragments of *VAR2CSA* gene (DBL 2X and DBL 3X) and four *vir* (*vir* 4, *vir* 12, *vir* 21 and *vir* 27) genes were analysed in this study.

### VAR2CSA-DBL2X and DBL3X domain

The genetic diversity of DBL 2X domain estimate on 537 sequences showed a π and Θω values of 0.08099 and 0.04907, respectively. The highest diversity was observed in Beninese isolates, while lowest diversity was seen in Colombian isolates. Despite the slight difference of *π* values, the same pattern was seen in all countries (Fig. 2).

The DBL3X domain from Malawi, Kenya and Mozambique depicted the same genetic diversity across this fragment. Almost superposed π variation curves were observed (Fig. 2). Genetic diversity ranged between 0.01766 and 0.13977 with the maximum amplitude of variation seen in Malawian isolates. Due to the high sequence diversity, a mutation point analysis was not performed for *VAR2CSA* gene.

### *Vir* 4

**Fig. 1:**
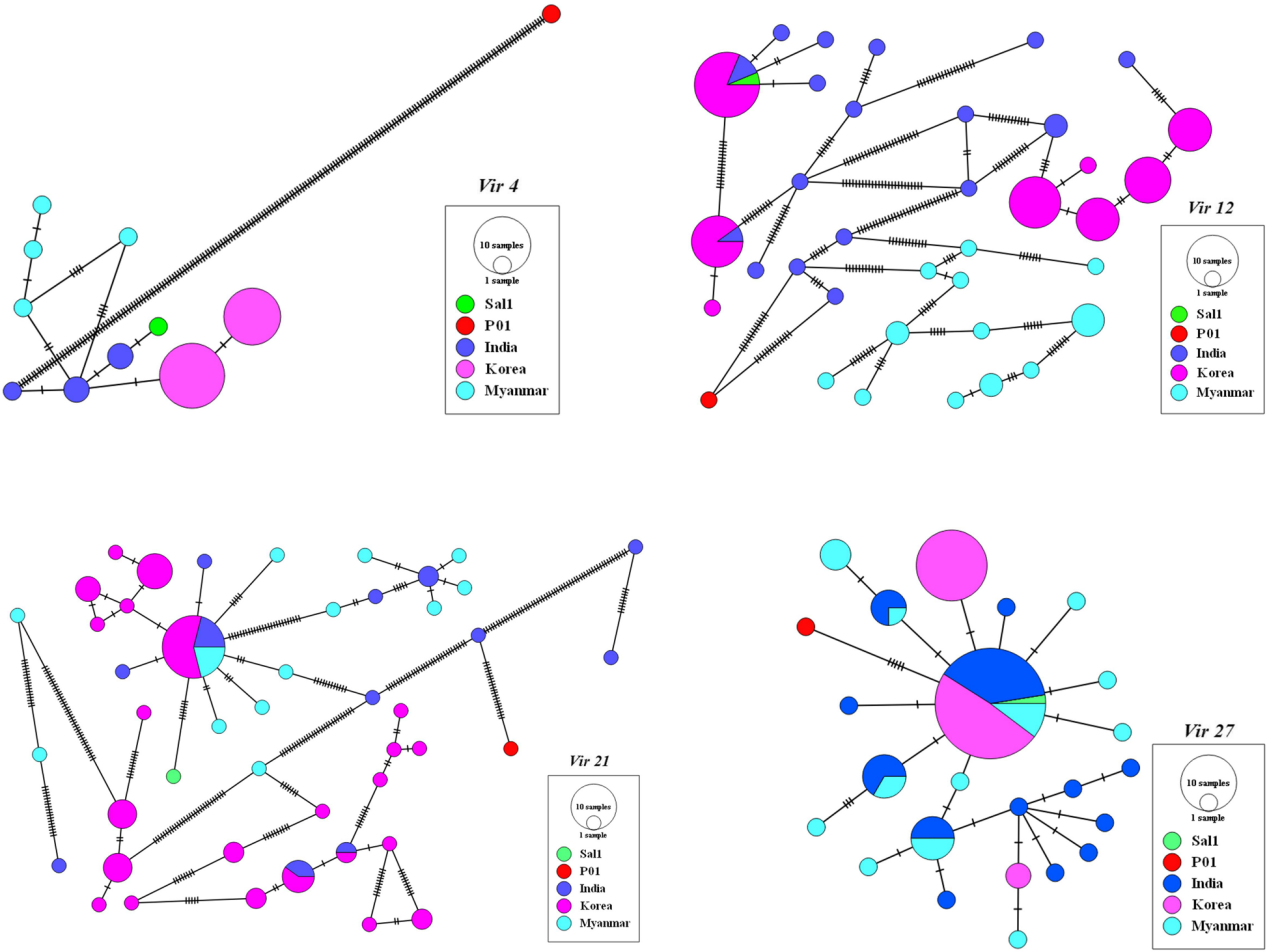
Minimum spanning tree network of *vir* haplotypes. The size of each circle is proportional to the given haplotype frequencies. The country of origin of each haplotype is represented by a specific colour. Two *P. vivax* Sal-I and PO01 reference sequences are also included for *vir* 4 (AAKM01000104 and PVP01_0006010), *vir* 12 (AAKM01000016 and PVP01_1035200), *vir* 21 (AAKM01000003 and PVP01_1101100) and *vir* 27 (AAKM01000041 and PVP01_0949900)

**Fig. 2:**
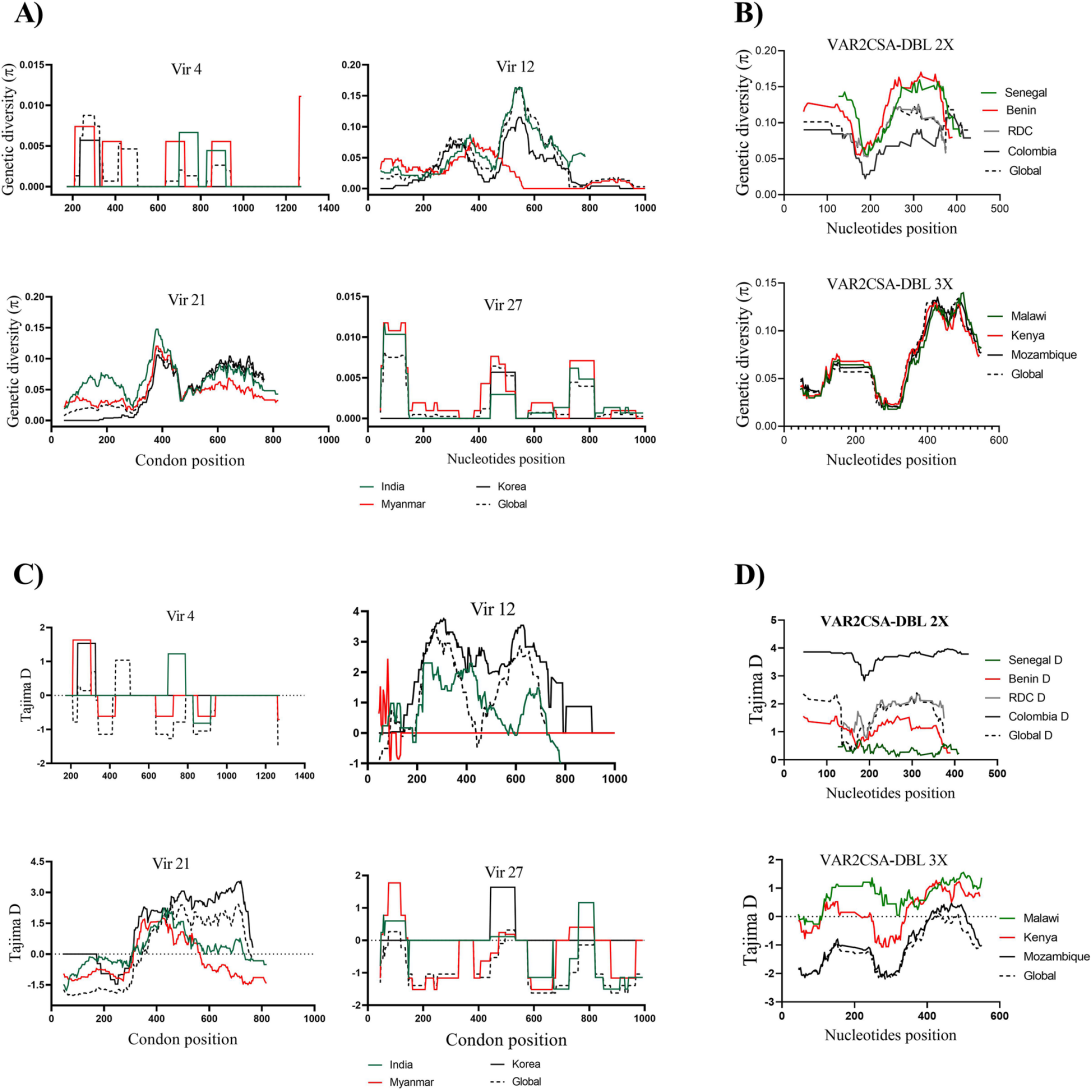
Sliding window plots analysis of nucleotide (nt) diversity (**A** and **B**) and Tajima’s D (**C** and **D**). The values are plotted on a sliding window of 90 bp and a step size of 3 bp

Nucleotide analysis showed that the average number of pairwise nucleotide difference (K) was 1.82. Nine *vir* 4 distinct haplotypes were found, and *Hd* was 0.748. All haplotypes were country specific, and none of them was identical to either the Sal I or PO01 reference strains (Fig. 1). The genetic diversity π and Θω values were 0.00171 and 0.00256, respectively (Table 1). Sliding window analysis of π with window length of 90 bp and step size of 3 bp showed that the diversity ranged from 0 to 0.00878 with the highest and lowest diversity in isolates from Myanmar and Korea, respectively (Fig. 2).

**Table 1:** Summary of polymorphism and neutrality test of *vir* and *var* gene

	**Fragment**	**N**	**S**	**K**	**H**	**Hd**	**π**	**Θω**	**Tajima's D**
***Vir***	*Vir 4*	32	11	1.82	9	0.748	0.00171	0.00256	-1.0632
	*Vir 12*	88	131	41.98	33	0.935	0.05597	0.03460	1.5906
	*Vir 21*	85	171	38.84	40	0.892	0.05077	0.04458	0.2578
	*Vir 27*	91	23	1.94	24	0.814	0.00187	0.00436	-1.7026
***Var2CSA***	DBL2X	537	99	23.81	446	0.996	0.08099	0.04907	1.2239
	DBL3X	315	307	34.31	298	0.999	0.06584	0.09312	-1.2702

S, number of polymorphic (segregating) sites; K, average number of nucleotide differences; π, pairwise nucleotide diversity; H, number of haplotypes; Hd, haplotype diversity; D, Tajima's D test

**Fig. 3:**
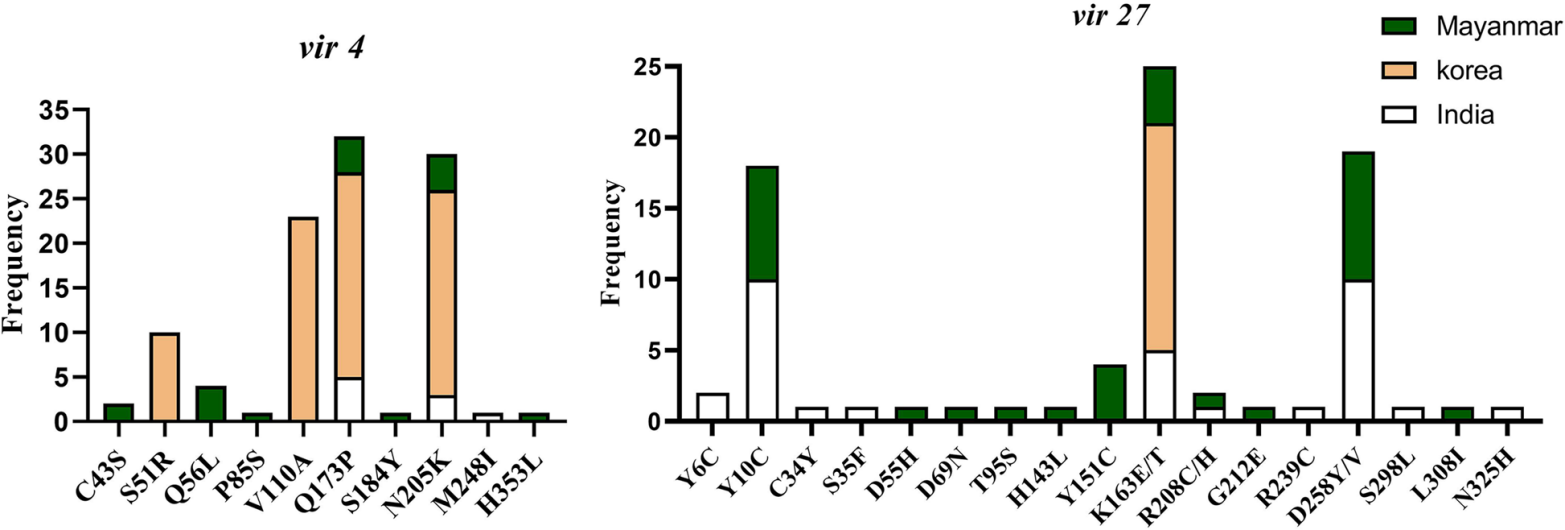
Frequencies distribution of mutations found in global *vir* 4 and *vir* 27 genes. Mutations Q173P and N205K in *vir* 4 were the most abundant and present in Myanmar, Korea and India. Mutation K163E/T was the most identified in *vir* 27 in Myanmar, Korea and India

Compared with the Sal-1 reference sequence, global *vir* 4 gene sequences showed 10 nonsynonymous mutations codon, *viz* C43S, S51R, Q56L, P85S, V110A, Q173P, S185Y, N205K, M248I and H353L (Fig. 3). Two mutations (Q173P, N205K) were found in all three countries. Mutations C43S, Q56L, P85S, S185Y and H353L were only reported from Myanmar, M248I specific to India, while S51R and V110A were only seen in Korean isolates. Amino acid substitutions V110A and Q173P found in 71.87% of isolates were the most abundant.

### *Vir* 12

The genetic diversity of *vir* 12 ranged from 0.01339 to 0.16502, with the lowest diversity observed in the Myanmar isolates. The most abundant haplotype (Hap1: 18.18%), which is identical to the Sal I reference sequence, was identified in Korea and India but not in Myanmar. The second most represented haplotype (Hap17: 11.36%) is exclusively shared between Korea (10.22%) and India (1.14%) (Fig. 1).

The *vir* 12 gene exhibited several nucleotide insertions and deletions (Indels) ranging from 3 to 30. All indels were multiples of 3, and the majority were 15 nucleotides long. Insertions were located after nucleotides A564, C773, G801 and C921 in comparison with the CDS *vir* 12 gene sequences of Sal 1. The shortest insertion of only three nucleotides (GAA/TAT/GGA) was found at position 564 in Indian, Myanmar and Korean isolates. The longest insertion of 30 nucleotides was located at positions 801 and 928 in Indian and Myanmar samples, but not in Korean samples (Table 2). Two insertions of 15 and 30 nucleotides in length were found at positions 859 and 928 in Indian and Myanmar isolates, respectively.

**Table 2:** Insertion and deletion observed in *vir 12* and *vir 21* genes

	**Insertion/deletion**	**Location**	**Length** **(Nucleotides)**	**Sequences**	**Countries**
***Vir 12***	Insertion	564	3	GAA / TAT / GGA	India, Myanmar and Korea
		773	15	AAAACCTGCACCTGT	India and Myanmar
		801	15	GCAAAACCTGTAGCG	India, Myanmar and Korea
		801	30	GCAAAACCTGTAGCAGCAAAACCTGCAGAC	India and Myanmar
		921	15	GCAAAACCTGTAGCG	India
	Deletion	859	15	CGACAAAACCTGCAA	India and Myanmar
		928	30	CTGTAGCGGCAAAACCAGCACCGGGAGAAG	India and Myanmar
***Vir 21***	Insertion	768	27	AGGGTTTCTGAAAAACCAAATATAAAT/ AAGTTATCTGAAAACCCAAATATAAAT	Myanmar
		773	5	TTCTG/ ATCTG	Korea
	Deletion	152	3	GGC	India and Myanmar
		786	5	TTTCT	Korea

Position number are given according to the coding sequences of Sal I reference strain accession number AAKM01000016 for *vir 12* and AAKM01000003 for *vir 21*

### *Vir* 21

For the *vir* 21 gene, none of the haplotypes was identical to the Sal I reference sequence (see Fig. 1). A total of 40 haplotypes were found with an Hd of 0.892, and a *π* value range from 0.00836 to 0.11716. The *π* values display the same pattern across the gene, regardless of the origin of the samples. After excluding gaps and ambiguous positions, the nucleotide diversity values were 0.05077 and 0.04458 for π and Θω, respectively (Table 1).

In the *vir* 21 gene, two insertions and two deletions were found. At position 768, only Myanmar isolates showed an insertion fragment of 27 nucleotides with two variants. In Korean samples, two variant insertions of five nucleotides (TTCTG/ATCTG) were observed at position 773 (Table 2). Korean isolates with an insertion at position 773 also had a five nucleotides deletion (TTTCT) at nucleotides position 786. India and Myanmar samples shared the same deletion sequences (GGC) at position 152. No insertions were observed in *vir* 21 of Indian isolates.

### *Vir* 27

Analysis of 91 sequences of *vir* 21 gene revealed 24 unique haplotypes with a Hd of 0.814. Nucleotide diversity values were estimated as 0.00187 and 0.00436 for π and Θω, respectively (Table 1). The sliding window plot shows fluctuation of *π* values between 0 and 0.01177, with the lowest values observed in Korean isolates (Fig. 2).

The most abundant haplotype was identical to the Sal I reference sequence and was found in all three countries. Korea exclusively had two haplotypes (Hap 15 and Hap 17), while India and Myanmar had Hap7, Hap9 and Hap10 (Fig. 1). Seventeen di- and trimorphic amino acid substitutions were found, including Y6C, Y10C, C34Y, S35F, D55H, D69N, T95S, H143L, Y151C, K163E/T, R208C/H, G212E, R239C, D258Y/V, S298L, L308I and N325H (Fig. 3). The most abundant mutation was K163E/F, found in 27.47% of *vir* 27 and identified in Indian, Myanmar and Korean sequences. The Korean *vir* 27 sequences only had the substitution K163T mutation. India and Myanmar have shown six (Y6C, C34Y, S35F, R239C, S298L and N325H) and seven (D55H, D69N, T95S, H143L, Y151C, G212E, and L308I) exclusive mutations, respectively. Additionally, the amino acid substitutions Y10C and D258Y/V were relatively abundant, accounting for 19.78% and 20.87%, respectively. Notably, these substitutions were absent in the Korean samples as shown in Fig. 3.

### Natural selection inference

Neutrality tests were conducted to evaluate the influence of natural selection on the *vir* and *VAR2CSA* genes by estimating the Tajima’s D values across these genes. The Tajima’s D values were negative for the *vir* 4 (Taj D =  − 1.0632), *vir* 27 (Taj D =  − 1.7026) and DBL3X (Taj D =  − 1.2702) fragments and positive for the *vir* 12 (Taj D = 1.5906), *vir* 21 (Taj D = 0.2578) and DBL2X (Taj D = 1.2239) fragments (Table 1). Although none of these genes showed significant deviation from neutral evolution, a sliding window analysis identified significant positive Tajima’s D values, which coincide with peaks of π values (Fig. 2).

**Fig. 4:**
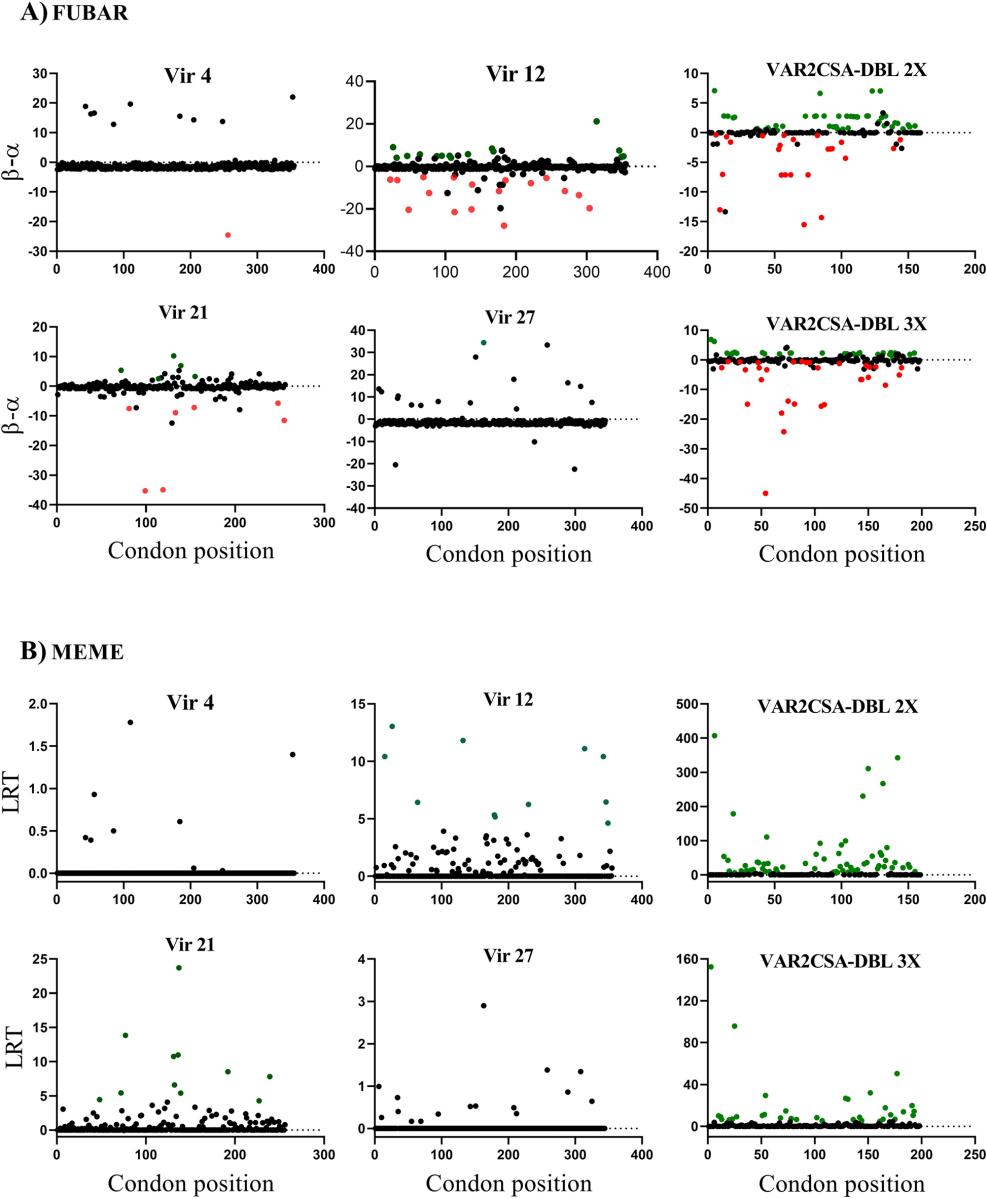
Summary of evolutionary process performed on the Datamonkey server for the detection of signature of departure from natural evolution. Two methods were used: Fast Unbiased Bayesian Approximation (FUBAR) and mixed effects model of evolution (MEME). The green and red dots represent codon positions identified as under diversifying and purifying selection, respectively. The red dots represent codon positions under negative/purifying selection identified by both methods. Black dots are potion that this shown any departure from natural evolution

**Fig. 5:**
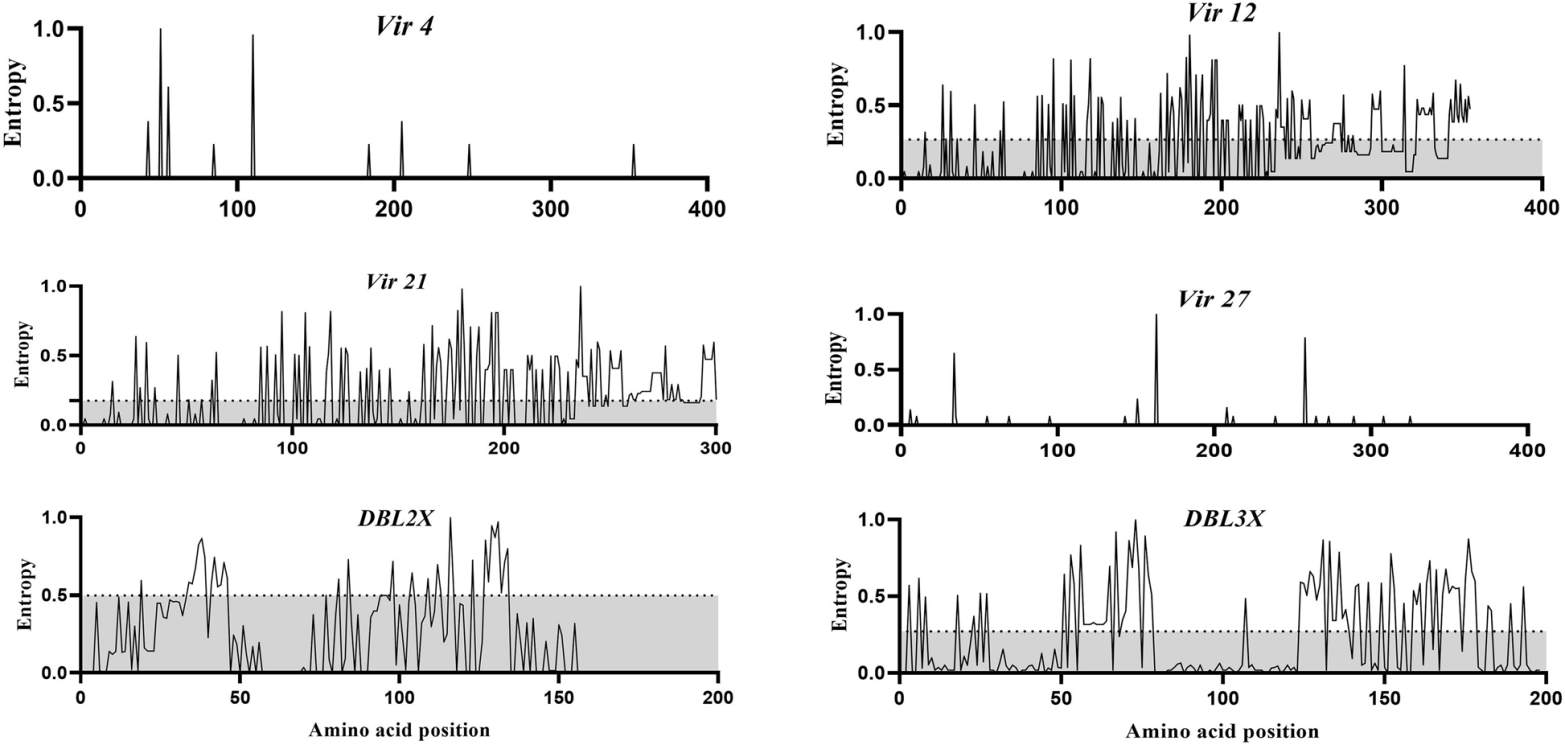
Normalized positional entropy across the vir (*vir* 4, *vir* 12, *vir* 21 and *vir* 27) and *VAR2CSA* (DBL2X and DBL3X fragment) genes. Positional entropy values were calculated using amino acid sequences for each gene. Dot line represents the average positional entropy. The *vir* 4 and *vir* 27 display the lowest entropy reflecting their conserved nature

**Fig. 6:**
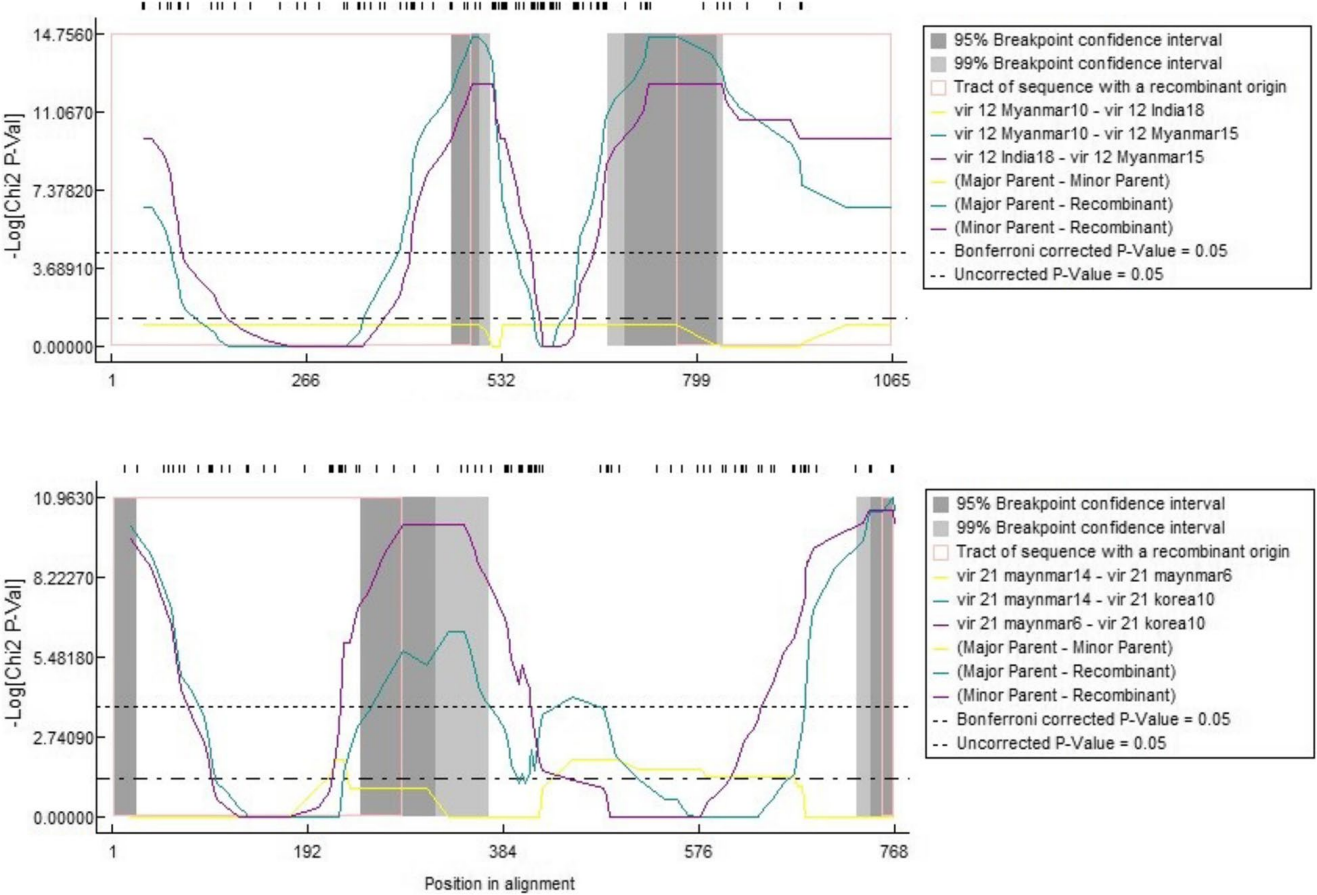
Recombination analysis results using RDP4. The plot shows the opposite log of chi-square *P*-value across the nucleotide sequences. The 95 and 99% breakpoint locations confidence intervals along with major/minor parent. Abd recombinants are shown

The MEME analysis of codon usage identified some individual codons under positive selection in the *vir* 12, *vir* 21, DBL2X and DBL3X fragments. FUBAR identified both positively and negatively selected codons scattered across the *vir* 12, *vir* 2 and *VAR2CSA* fragments 2X and 3X (Fig. 4). The analysis indicates that *vir* 12 and *vir* 21 genes are subject to higher evolutionary pressure than genes *vir* 4 and *vir* 27. This result is supported by the positional entropy results. The amino acid composition is almost completely conserved for *vir* 4 (*ΔH* = 0.007) and *vir* 12 (*ΔH* = 0.008), as shown by the FUBAR and MEME results (Fig. 5). On the contrary, there was a lower level of amino acid conservation in the *vir* 12 and *vir* 21 sequences, with average positional entropies of 0.266 and 0.176, respectively (Fig. 6).

### Recombination

To investigate potential recombination signals in global *vir* and *var* genes from Asia, seven algorithms were executed using RDP4 software. The recombination analysis identified nine and seven significant events in *vir* 12 and *vir* 21, respectively. The GARD algorithm located the breakpoints at positions 321, 498 and 675 in *vir* 12 and at positions 340 and 463 in *vir* 21, which is consistent with the range provided by RDP4 program (Supplementary file 2). The MaxChi model suggests that the *vir* 4 and *vir* 12 isolates from Myanmar are the major parents from which other isolates emerged as recombinants (Fig. 6 and Supplementary file 2). No recombination events were detected by either RDP4 or GARD in *vir* 4 and *vir* 27 genes.

Due to the high level of sequence heterogeneity in terms of size and nucleotide diversity, recombination analysis could not be performed for *VAR2CSA* gene.

## Discussion

There is mounting evidence that *Plasmodium* VSAs families are not exclusively associated with blood-stage infection but also play a potential role in various mechanisms throughout the parasite’s life cycle, making them suitable targets for vaccine development studies [22, 23]. Currently, two gestational malaria vaccine candidates, based on fragments of the *VAR2CSA* protein from 3D7 and FCR3 reference strains, are in phase Ia/b clinical trials [24]. The gene is used as a vaccine target due to its relatively conserved sequence compared to other genes in the same family, though its genetic diversity remains 500-fold higher than that of random housekeeping genes in *P. falciparum* [25]. The genetic diversity of DBL2X (*π* = 0.08099; *Θw* = 0.04907) and DBL3X (*π* = 0.06584; *Θw* = 0.09312) fragments was higher compared to the studied *vir* genes. The genetic diversity patterns and Tajima’s D sliding window were almost identical, particularly for the DBL3X fragment. Although Tajima’s D values varied between countries, they showed the same pattern across the gene, indicating the presence of convergent molecular selection that facilitated the evolution of the *Plasmodium* parasite [26]. The *VAR2CSA* gene contains several codons that are under positive and negative selection pressure, indicating the immune pressure on DBL2X and DBL3X fragments, which have been used as vaccine targets [27, 28].

Additionally, we analysed the evolutionary pattern of the *vir* genes, including *vir* 4, *vir* 12, *vir* 21 and *vir* 27, in *P. vivax* field isolates from India, Myanmar and Korea. The highest genetic diversity values were observed in *vir* 12 (*π* = 0.05597; *Θω* = 0.03460) and *vir* 21 (*π* = 0.05077; *Θω* = 0.04458). This genetic diversity was accentuated by a large number of insertions and deletions up to 30 nucleotides long. The diversification of these genes is strongly associated with random recombination, particularly facilitated by their sub-telomeric localization [23, 29]. In contrast, *vir* 4 and *vir* 27 exhibit a higher degree of conservation, with only 1.02% and 2.21% of segregating sites across the gene, respectively. The low diversity and negative Tajima’s D values for *vir* 4 (*π* = 0.00171; TajD =  − 1.0632) and *vir* 27 (*π* = 0.00187; TajD =  − 1.7026) suggest a decrease in polymorphism, possibly due to purifying selection of unfavourable haplotypes that are purged from the genetic pool and/or a population expansion [30].

Most SNPs and indel observed in *vir* genes were not exclusive to any particular country. Also, the global patterns of genetic diversity and of Tajima’s D sliding window values were remarkably similar, indicating comparable demographic histories of these populations, but also that these genes are subject to similar molecular mechanisms pressure. The available evidence suggests a potential relationship between *P. vivax* parasite in these countries, but further studies on *vir* genes are needed to draw a strong conclusion. The higher number of SNPs in *vir* 4 and *vir* 27 among isolates from Myanmar compared to those from India and Korea suggests a higher evolutionary pressure driving genetic diversity in this region [19].

Recombination events were assessed prior to inferring selective pressure. Only three and two breakpoints were identified in the *vir* 12 and *vir* 21 genes, respectively, which would have little effect on the identification of codons under diversifying or purifying selection. No recombination even was observed in *vir* 4 and *vir* 27 indicating their conserved nature and suitability as target for *P. vivax* vaccine development.

Although the implication and mechanism of action of *vir* gene superfamilies have not been elucidated yet, some studies have suggested their involvement in *P. vivax* malaria pathogenesis [13, 31]. Several studies from different countries with diverse endemicity levels have reported the immunogenic properties of *vir* antigens in pregnant and nonpregnant women [11, 29, 32, 33]. The high rate of amino acid substitution, insertion and deletions in *vir* 12 and *vir* 21, indicated by their high entropy values, may have impacted the antigenicity of *P. vivax* facilitating the parasite’s escape from the host’s immune system [34]. These two genes are under significant immune pressure, as evidenced by multiples codons under positive or negative selection. For their adaptation under harmful conditions and survival, parasites are able to undergo positive and negative selection simultaneously [35, 36]. Positive selection generates diversification of advantageous genetic variants, while negative selection leads to genetic conservation. The main driver of adaptive evolution is positive natural selection. This refers to the tendency for advantageous traits to become more prevalent in a population. In the context of host–pathogen coevolution, such as in the case of human *Plasmodium*, the pathogen is under constant pressure to develop new strategies to survive within the host and evade the immune system’s defences [37]. The majority of sites under selection pressure exhibit positive rather than negative selection, indicating that most sites are diversified for adaption, and only a few are conserved.

Research has shown that an effective vaccine should contain a protein fragment with minimal allelic forms that are shared by global parasite populations and maintained through balanced selection. The analysis of *vir* and *var* genes in this study indicates that they are all under balancing selection, which supports their potential as vaccine candidates. A large-scale genetic polymorphism analysis including extensive sample from diverse geographic origin is a prerequisite for the consideration of given gene as a vaccine candidate target. However, unlike the extensively studied *var* genes, there have been limited studies on the genetic polymorphisms of *vir* genes, making it difficult to appreciate their global genetic diversity. To create a comprehensive genetic diversity pattern of *vir* genes that can be used for vaccine design, further studies are necessary.

## Conclusion

In this study, we analysed genetic diversity and evolutionary pattern of two fragments (DBL2X and DBL3X) of *VAR2CSA* gene and four *vir* genes (*vir* 4, *vir 12*, *vir* 21 and *vir* 27). Among the *vir* genes studied, *vir* 4 and *vir* 27 were more conserved, whereas *vir* 12 and *vir* 21 were highly diverse. Similarly, *VAR2CSA* depicted higher genetic diversity. Overall, these genes are likely to be under balancing selection, although some specific codons were under positive or negative selection pressure. Further extensive studies are required to draw a clear picture of genetic pattern of the *vir* gene family in terms of evolution, as very little data is available. For both *vir* and *var* genes, functional experiments based on the genetic results are needed to determine the most relevant allelic forms to include in a vaccine formulation to induce a broad immune response.

### Limitations and challenges

Despite the usefulness of this work, it is important to point out some limitations that pose challenges in the complex study of VSA-coding genes. Firstly, only amplicons from field isolates were analysed, thus not including whole genome sequencing data, which provide a broader understanding of evolutionary genetics of *var* and *vir* genes. Secondly, the biological mechanism of mutually exclusive expression of the 60 *var* genes in *P. falciparum* and their implication in pathogenesis is not yet elucidated [22]. Similarly, only a small number of *vir* genes have been studied by a limited number of authors so far [23]. Only 10 *vir* genes out of > 1200 identified have been studied genomically or immunologically. This lack of conclusive data on the involvement of *var* and *vir* genes has made it difficult to the understand their mechanisms of action and implication in malaria pathophysiology [23, 23]. The genetic significance of *var* and *vir* gene polymorphisms in malaria pathogenesis need to be further investigated to overlay with genomic data and ultimately translated into new alternative malaria control strategies.

### Supplementary Information

Supplementary Material 1:** Supplementary file 1. Table 1.** *Vir *and* Var2CSA* sequences included in the study. **Supplementary file 2.** Recombination analysis performed in RDP4 with default parameters. *Vir 12. Vir 21*. Breakpoints confirmed by GARD algorithm.
