# Supplementary material for: Genetic diversity and natural selection analysis of VAR2CSA and vir genes: implication for vaccine development
Source: Genomics Inform. 2024 Jul 15;22:11. doi: 10.1186/s44342-024-00009-0 (PMC11247734; doi:10.1186/s44342-024-00009-0)
Supplement: Supplementary file 1 — Supplementary Material 1: Supplementary file 1. Table 1. Vir and Var2CSA sequences included in the study. Supplementary file 2. Recombination analysis performed in RDP4 with default parameters. Vir 12. Vir 21. Breakpoints confirmed by GARD algorithm. [file 44342_2024_9_MOESM1_ESM.docx]

**Supplementary materials**

**Supplementary file 1:** *Vir* and *Var2CSA* sequences included in the study.

| Vir gene | N | Country | n | Accession number |
| --- | --- | --- | --- | --- |
| *Vir 4* | 32 | India | 5 | JQ733948 - JQ733952 |
|  |  | Korea | 23 | KY608341 - KY608363 |
|  |  | Myanmar | 4 | MN436008 - MN436011 |
| *Vir 12* | 88 | India | 19 | JQ733953 - JQ733971 |
|  |  | Korea | 52 | KY608364 - KY608419 |
|  |  | Myanmar | 17 | MN436012 - MN436028 |
| *Vir 21* | 85 | India | 17 | JQ733972 - JQ733988 |
|  |  | Korea | 52 | KY608420 - KY608471 |
|  |  | Myanmar | 16 | MN436029 - MN436044 |
| *Vir 27* | 91 | India | 33 | JQ733915 - JQ733947 |
|  |  | Korea | 35 | KY608472 - KY608506 |
|  |  | Myanmar | 23 | MN436045 - MN436067 |
| VAR2CSA  (DBL2X) | 537 | Senegal | 24 | GQ358101- GQ358105  GQ358108- GQ358113  GQ358106- GQ358121  GQ358123- GQ358128  GQ358135 |
|  |  | Benin | 62 | KT359638- KT359649; KT35963854- KT359656  KT359658, KT359662, KT359667- KT359669  KT359672, KT359674- KT359679 KT359681- KT359692; KT359695, KT359697- KT359700  KT359705- KT359707; KT359709; KT359712, KT359713; KT359715- KT359723  KT359725- KT359727 |
|  |  | Democratic republic of Congo | 405 | MG029761, MG029763, MG029766, MG029768, MG029770, MG029761- MG029806  MG029808- MG02909, MG029911- MG02914  MG029918, MG029920, MG029761, MG029922-MG02998, MG030000- MG030042, MG030060-MG030104, MG030177- MG030203, MG030337- MG030402, |
|  |  | Colombia | 46 | MF1090063- MF1090108 |
| VAR2CSA  (DBL3X) | 315 | Malawi | 20 | AY461586- AY461586, DQ286630, DQ286631  DQ306266, DQ306269- DQ306272, DQ306282, DQ306288, DQ306289, DQ306294; DQ306298 |
|  |  | Kenya | 68 | JN615483- JN615524; JN615527,  JN615530 -JN615555 |
|  |  | Mozambique | 218 | JQ780017- JQ780047  JQ440176- JQ440285, JQ440287- JQ440322 JQ440324-JQ440362 |
|  |  | Mali | 3 | MN631060- MN631062 |

**Supplementary file 2**: Recombination analysis performed in RDP4 with default parameters

- ***Vir 12***


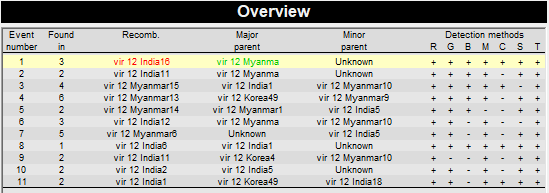


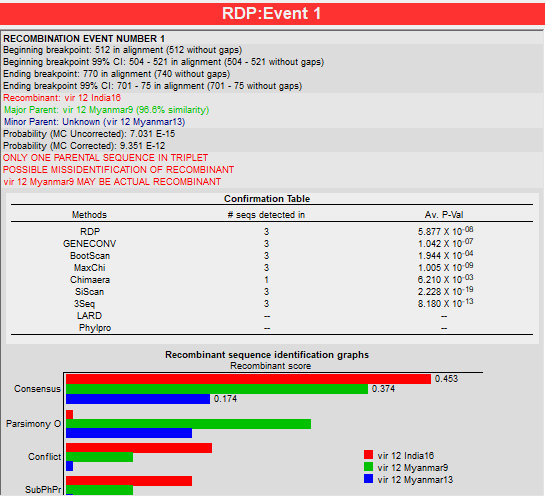

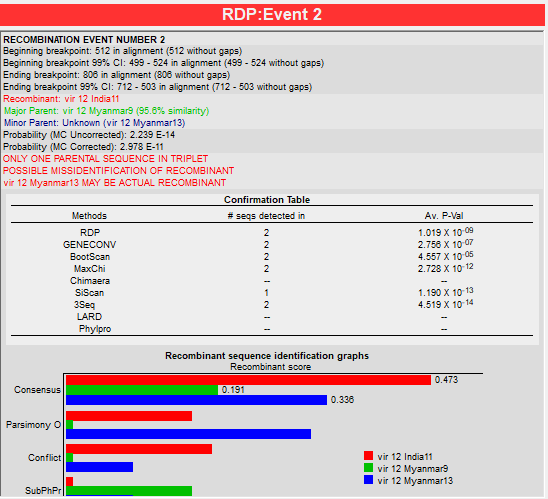


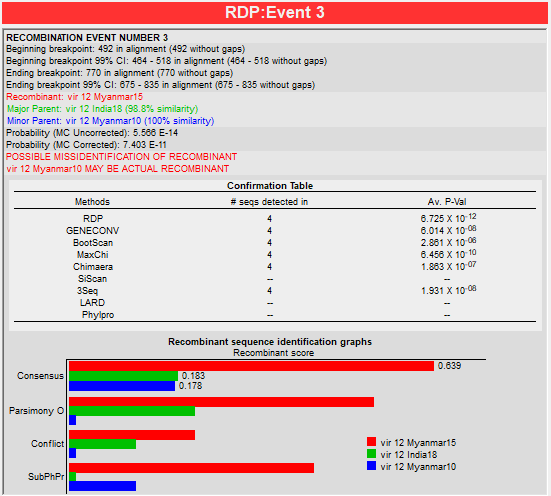

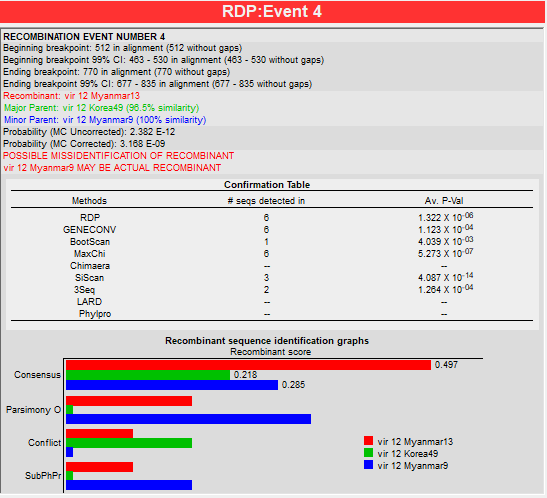


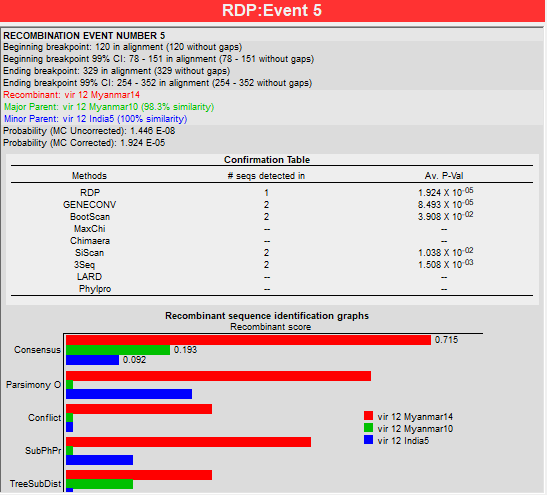

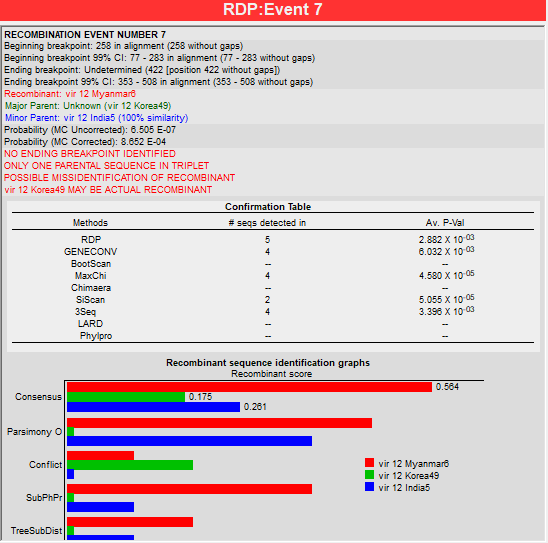


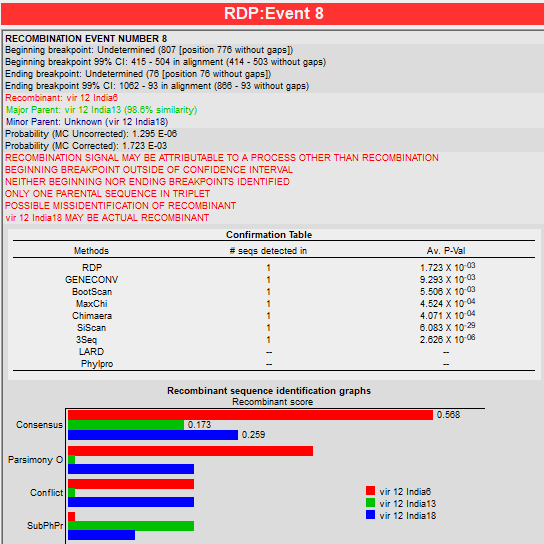

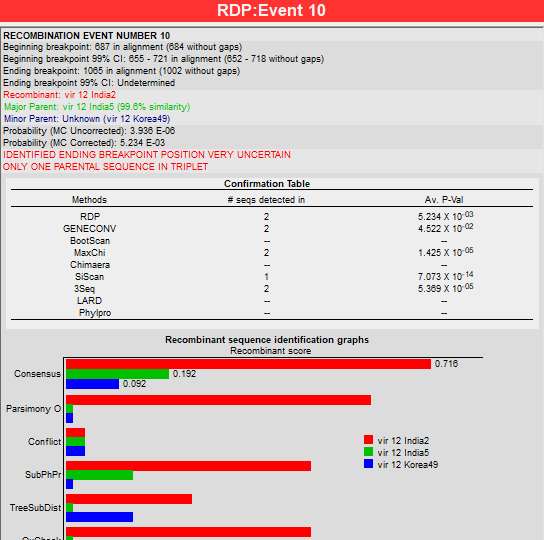


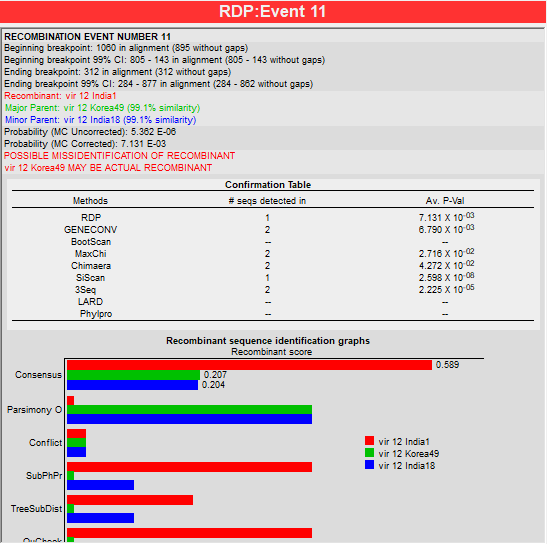


- ***Vir 21***


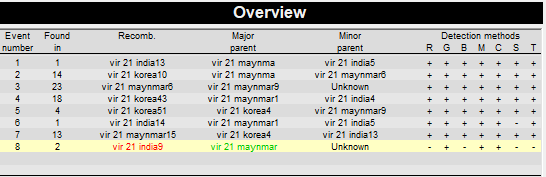


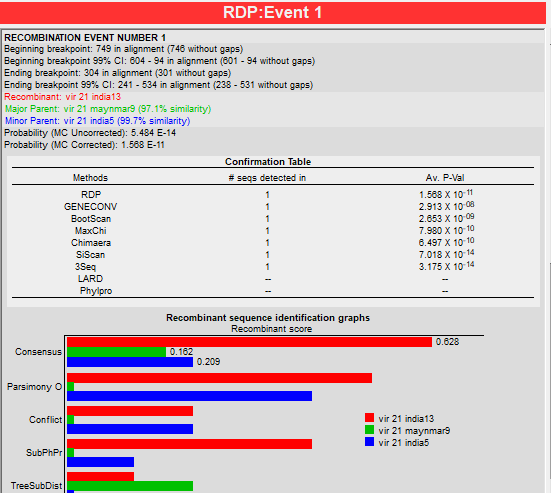

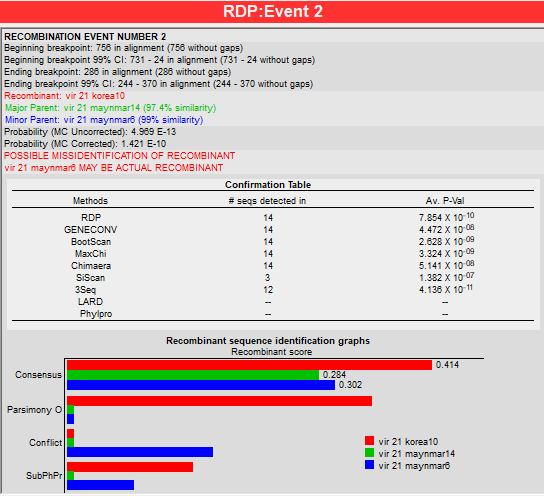


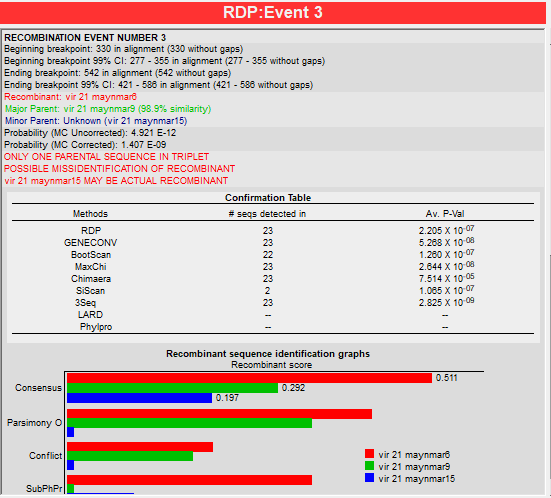

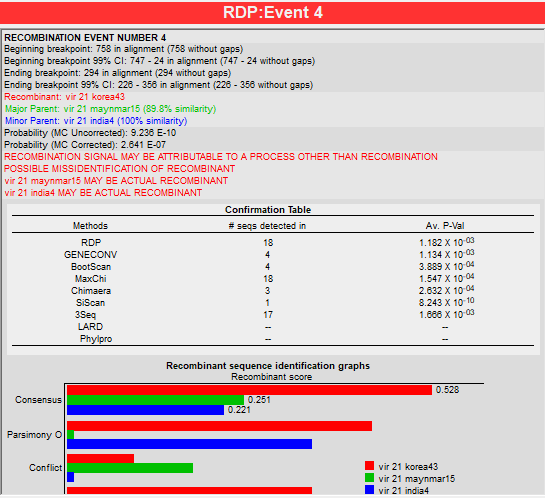


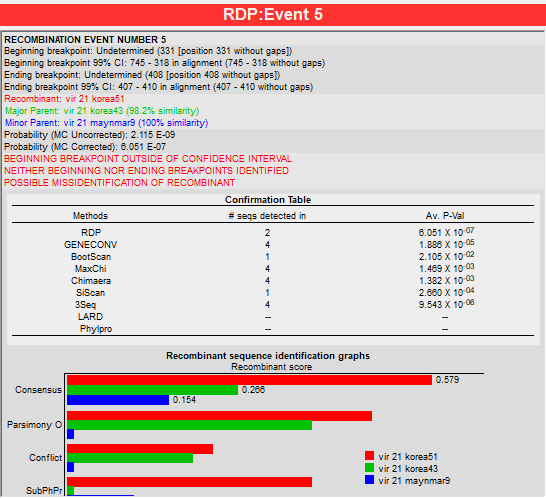

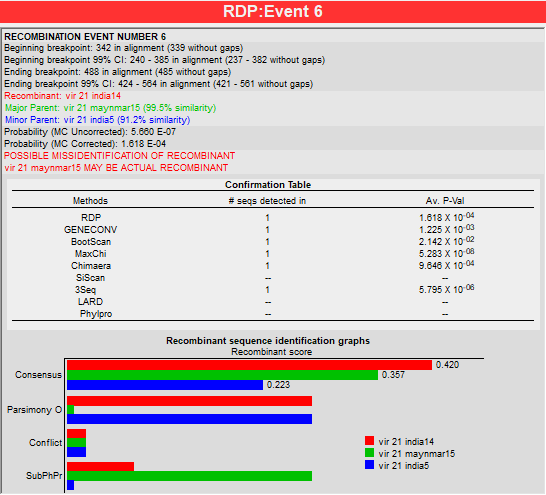


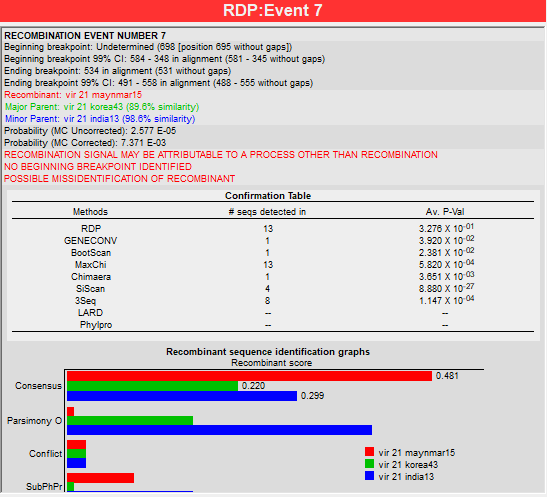


**Breakpoints confirmed by GARD algorithm**

| **Gene** | **Δ c-AIC vs the null model** | **Δ c-AIC vs the single tree multiple partition** | **Evident breakpoint** | **Position** |
| --- | --- | --- | --- | --- |
| Vir 12 | 1498.08 | 1381.42 | 3 | 321; 498; 675 |
| Vir 21 | 720.987 | 633.449 | 2 | 340; 463 |
